# IoT-Based Applications in Healthcare Devices

**DOI:** 10.1155/2021/6632599

**Published:** 2021-03-18

**Authors:** Bikash Pradhan, Saugat Bhattacharyya, Kunal Pal

**Affiliations:** ^1^Department of Biotechnology and Medical Engineering, National Institute of Technology, Rourkela 769008, India; ^2^School of Computing,Engineering & Intelligent System, Ulster University, Londonderry, UK

## Abstract

The last decade has witnessed extensive research in the field of healthcare services and their technological upgradation. To be more specific, the Internet of Things (IoT) has shown potential application in connecting various medical devices, sensors, and healthcare professionals to provide quality medical services in a remote location. This has improved patient safety, reduced healthcare costs, enhanced the accessibility of healthcare services, and increased operational efficiency in the healthcare industry. The current study gives an up-to-date summary of the potential healthcare applications of IoT- (HIoT-) based technologies. Herein, the advancement of the application of the HIoT has been reported from the perspective of enabling technologies, healthcare services, and applications in solving various healthcare issues. Moreover, potential challenges and issues in the HIoT system are also discussed. In sum, the current study provides a comprehensive source of information regarding the different fields of application of HIoT intending to help future researchers, who have the interest to work and make advancements in the field to gain insight into the topic.

## 1. Introduction

In recent years, the healthcare industry has shown rapid growth and has been a major contributor to revenue and employment [1]. A few years ago, the diagnosis of diseases and abnormality in the human body was only being possible after having a physical analysis in the hospital. Most of the patients had to stay in the hospital throughout their treatment period. This resulted in an increased healthcare cost and also strained the healthcare facility at rural and remote locations. The technological advancement that has been achieved through these years has now allowed the diagnosis of various diseases and health monitoring using miniaturized devices like smartwatches. Moreover, technology has transformed a hospital-centric healthcare system into a patient-centric system [2, 3]. For example, several clinical analyses (such as measuring blood pressure, blood glucose level, pO_2,_ level, and so on) can be performed at home without the help of a healthcare professional. Further, the clinical data can be communicated to healthcare centers from remote areas with the help of advanced telecommunication services. The use of such communication services in conjunction with the rapidly growing technologies (e.g., machine learning, big data analysis, Internet of things (IoT), wireless sensing, mobile computing, and cloud computing) has improved the accessibility of the healthcare facilities.

IoT has not only enhanced the independence but also diversified the ability of the human to interact with the external environment. IoT, with help of futuristic protocol and algorithms, became a major contributor to global communication. It connects a large number of devices, wireless sensors, home appliances, and electronic devices to the Internet [4]. The application of IoT can be found in the field of agriculture [5], automobiles [6, 7], home [8], and healthcare [1, 9]. The growing popularity of the IoT is due to its advantage of showing higher accuracy, lower cost, and its ability to predict future events in a better way. Further, increased knowledge of software and applications, with the upgradation of mobile and computer technologies, easy availability of wireless technology, and the increased digital economy have added to the rapid IoT revolution [10]. The IoT devices (sensors, actuators, and so on) have been integrated with other physical devices to monitor and exchange information using different communication protocols such as Bluetooth, Zigbee, IEEE 802.11 (Wi-Fi), and so on. In healthcare applications, the sensors, either embedded or wearable on the human body, are used to collect physiological information such as temperature, pressure rate, electrocardiograph (ECG), electroencephalograph (EEG), and so on [11] from the patient's body. Additionally, environmental information such as temperature, humidity, date, and time can also be recorded. These data help in making meaningful and precise inferences on the health conditions of the patients. Data storage and accessibility also play an important role in the IoT system as a large amount of data are acquired/recorded from a variety of sources (sensors, mobile phones, e-mail, software, and applications). The data from the aforesaid sensing devices are made available to doctors, caregivers, and authorized parties. The sharing of these data with the healthcare providers through cloud/server allows quick diagnosis of the patients and medical intervention if necessary. The cooperation between the users, patients, and communication module is maintained for effective and secure transmission. Most of the IoT systems use a user interface that acts as a dashboard for medical caregivers and performs user control, data visualization, and apprehension. An ample amount of research has been discovered in the literature that has reported the progress of the IoT system in healthcare monitoring, control, security, and privacy [12]. These accomplishments illustrate the effectiveness and propitious future of IoT in the healthcare sector. However, the main concern while designing an IoT device is maintaining the quality of service matrices that include privacy of information sharing, security, cost, reliability, and availability.

Intending to maximize the employability of IoT in healthcare systems, many countries have adopted new technology and policies. This transformed the current research in the healthcare sector into a more beneficial field to explore. The motivation of this paper is to summarize the advancement of state-of-the-art studies in IoT-based healthcare systems and to provide a systematic review of its enabling technologies, services, and applications.

## 2. Architecture of Healthcare IoT (HIoT)

**Figure 1:**
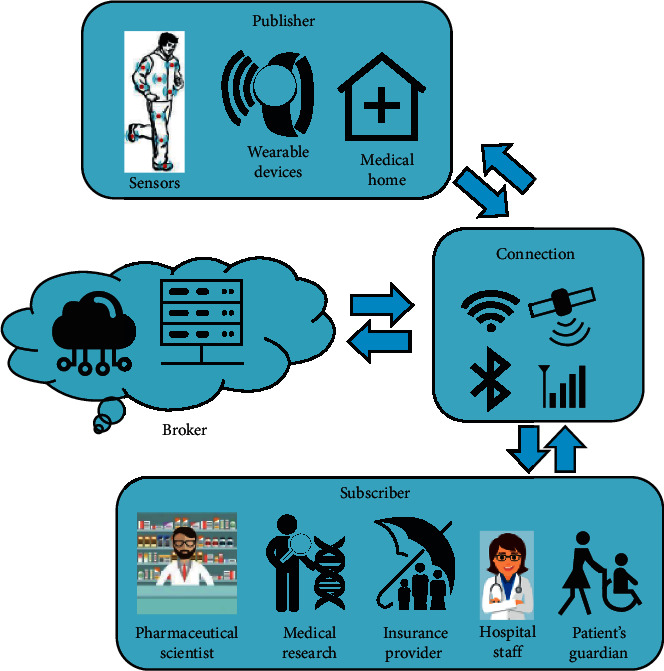
Architecture of an HIoT framework (reproduced from [13] under Creative Commons License).

The framework of the IoT that is applied for healthcare applications aids to integrate the advantages of IoT technology and cloud computing with the field of medicine. It also lays out the protocols for the transmission of the patient's data from numerous sensors and medical devices to a given healthcare network. The topology of an HIoT is the arrangement of different components of an IoT healthcare system/network that are coherently connected in a healthcare environment. A basic HIoT system contains mainly three components (Figure 1) such as publisher, broker, and subscriber [14]. The publisher represents a network of connected sensors and other medical devices that may work individually or simultaneously to record the patient's vital information. This information may include blood pressure, heart rate, temperature, oxygen saturation, ECG, EEG, EMG, and so on [13]. The publisher can send this information continuously through a network to a broker. The broker is responsible for the processing and storage of the acquired data in the cloud. Finally, the subscriber indulges in the continuous monitoring of the patient's information that can be accessed and visualized through a smartphone, computer, tablet, etc. Herein, the publisher can process these data and give feedback after the observation of any physiological anomaly or degradation in the patient's health condition. The HIoT assimilates discrete components into a hybrid grid where a specific purpose is dedicated to each component on the IoT network and cloud in the healthcare network. Since the topology for an HIoT depends on the healthcare demand and application, it is hard to suggest a universal structure for HIoT. Numerous structural changes have been adopted in the past for an HIoT system [15–17]. It is crucial to list out all associated activities related to the desired health application while designing a new IoT-based healthcare system for real-time patient monitoring. The success of the IoT system depends on how it is satisfying the requirements of healthcare providers. Since each disease needs a complex procedure of healthcare activities, the topology must follow the medical rules and steps in the diagnosis procedure.

## 3. HIoT Technologies

**Figure 2:**
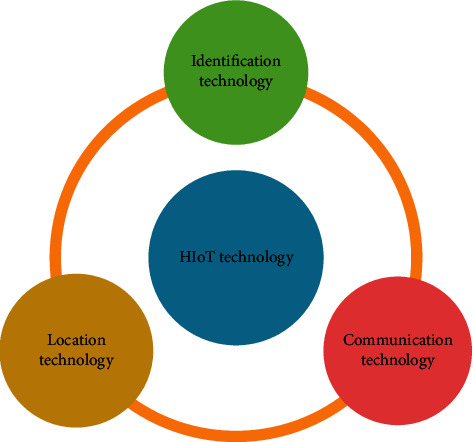
Classification of IoT technology.

The technologies that are used to develop an HIoT system is crucial. This is because the use of specific technology can enhance the ability of an IoT system [18]. Hence, to integrate different healthcare applications with an IoT system, various state-of-the-art technologies have been adopted. These technologies can broadly be categorized into three groups, namely, identification technology, communication technology, and location technology (Figure 2).

### 3.1. Identification Technology

A practical consideration in designing an HIoT system is the accessibility of the patient's data from the authorized node (sensor), which may be present at remote locations. This can be carried out with effective identification of the nodes and sensors that are present in the healthcare network. Identification follows the process of assigning a unique identifier (UID) to each authorized entity so that it can be easily identified and unambiguous data exchange can be achieved. In general, every resource associated with the healthcare system (hospital, doctor, nurses, caregivers, medical devices, and so on) is accompanied by a digital UID [19]. This ensures the identification of the resources as well as the connection among the resources in a digital domain. In the literature, numerous standards for identification have been reported [20]. The Open Software Foundation (OSF) has developed two different identifiers, namely, a universally unique identifier (UUID) and a globally developed unique identifier (GUID). UUID, a part of Distributed Computing Environment (DCE), can be operated without the requirement of centralized coordination [21]. In a healthcare network, the sensors and actuators are identified and addressed separately which helps in the proper functioning of the system. However, there may be a chance that the unique identification of a component may change throughout the life cycle of the IoT system due to the continuous upgradation of the IoT-based technologies. Hence, the device must have a provision to update this information to maintain the integrity of the healthcare device/system. This can be reasoned to the fact that the change in the configuration not only affects the process of tracking the network component(s) but also may result in a flawed diagnosis. Additionally, the application of IoT in healthcare demands new technologies that have the capability to (1) locate things based on a global identification number, (2) safely manage the identity of the components using different encryption and authentication techniques, and (3) build a global directory search for efficient discovery of IoT services under the UUID scheme.

### 3.2. Communication Technology

Communication technologies ensure the connection among different entities in an HIoT network. These technologies can be broadly divided into short-range and medium-range communication technology. The short-range communication technologies are the protocols that are used to establish a connection among the objects within a limited range or a body area network (BAN), whereas the medium-range communication technologies usually support communication for a large distance, e.g., communication between a base station and the central node of a BAN. The distance of communication may vary from a few centimeters to several meters in the case of short-range communication. In most of the HIoT applications, short-range communication technology is preferred. Some of the most widely used communication techniques include RFID, Wi-Fi, Zigbee, Bluetooth, etc.

*Radio-Frequency Identification (RFID)*. RFID is used for short-range communication (10 cm–200 m). It consists of a tag and a reader. The tag is developed using a microchip and antenna. It is used to uniquely identify an object/device (healthcare equipment) in the IoT environment. The reader transmits or receives information from the object by communicating with a tag using radio waves. In the case of IoT, the data used in the tag are in the form of an electronic product code (EPC). RFID enables healthcare providers to quickly locate and track healthcare equipment. The main advantage of RFID is that it does not need an external power source. However, it is a highly insecure protocol and may show compatibility issues while connecting with a smartphone.

*Bluetooth*. Bluetooth is also a short-distance wireless communication technology that uses UHF (ultra-high frequency) radio waves. This technology allows wireless connection between two or more medical devices. The frequency range of Bluetooth is 2.4 GHz. The Bluetooth protocol presents a communication range of up to 100 m. Bluetooth gives data protection in the form of authentication and encryption. The advantage of Bluetooth lies in its low cost and energy efficiency. It also ensures a lower interference among the connected devices during data transmission. However, when the healthcare application demands long-range communication, this technology fails to meet the requirement.

*Zigbee*. Zigbee is one of the standard protocols that interconnects medical devices and transmits information back and forth. The frequency range of Zigbee is similar to Bluetooth (2.4 GHz). However, it possess a higher communication range than that of Bluetooth devices. This technology adopts a mesh network topology. It consists of end nodes, routers, and a processing center. The processing center is responsible for data analysis and aggregation. The mesh network ensures uninterrupted connection among other devices even when there is a fault in one or two devices. The advantages of Zigbee lies in its low power consumption, high transmission rate, and high network capacity.

*Near-Field Communication (NFC)*. The basic concept of NFC is the electromagnetic induction between the two-loop antennas that are placed near to each other. This technology is similar to RFID that also uses electromagnetic induction for data transmission. The NFC devices can be operated in two modes: active and passive. In the case of passive mode, only one device generates the radiofrequency while the other device acts as a receiver. In the case of active mode, both devices can produce the radiofrequency simultaneously and can transmit data without pairing [22]. The main advantages of NFC are its easy operability and an efficient wireless communication network. However, it is applicable for a very short range of communication.

*Wi-Fi*. Wireless Fidelity (Wi-Fi) is a wireless local area network (WLAN) that follows the IEEE 802.11 standard. It provides a higher transmission range (within 70 ft.) as compared to Bluetooth. Wi-Fi builds a network very quickly and easily. Hence, it is mostly used in hospitals. The wide application of Wi-Fi lies in its easy compatibility with smartphones and its provision to support robust security and control. However, it shows a relatively higher power usage and the network performs inconsistently.

*Satellite*. Satellite communication is found to be more effective and beneficial in remote and widely separated geographical areas (such as rural areas, mountains, peaks, oceans, and so on) where other modes of communication cannot reach easily. The satellite receives signals from the land, amplifies those signals, and then resends them to Earth. More than 2000 satellites are orbiting around the Earth. The advantage of satellite communication technology includes high-speed data transfer, instant broadband access, stability, and compatibility of the technology. However, the power consumption associated with satellite communication is very high as compared to other communication techniques.

### 3.3. Location Technology

The real-time location system (RTLS) or location technologies are used to identify and track the position of an object within the healthcare network. It also tracks the treatment process based on the distribution of available resources. One of the most widely used technologies is the Global Positioning System, which is commonly known as GPS. It makes use of satellites for tracking purposes. An object can be detected through GPS as long as there exists a clear line of sight between the object and four different satellites. In HIoT, it can be employed to detect the position of the ambulance, healthcare provider, caregivers, patients, etc. However, the application of GPS is only limited to outdoor applications as the surrounding infrastructures can act as an obstruction to the communication between the object and the satellite. In such cases, a local positioning (LPS) network can be effectively used. LPS can track an object by sensing the radio signal that is emitted from the traveling object to an array of predeployed receivers [23]. Various short-distance communication technologies such as RFID, Wi-Fi, Zigbee, and so on can also be used to employ LPS. However, ultra-wideband (UWB) radio is preferred due to its advantage of higher temporal resolution. This enables the receiver to accurately measure the arrival time. Young [24] and Zetik [25] have employed UWB-based localization system that uses the time difference of arrival (TDOA) for tracking. In the literature, other measuring parameters have also been reported in designing a UWB-based localization system such as relative and differential time of arrrival [26], round trip time of flight [20], and so on. GPS, along with the different high bandwidth communication technologies, may be explored in the future to develop smart healthcare networks.

## 4. Services and Application of HIoT

The recent advancement in the IoT technology has enabled the medical devices to make real-time analysis that was not possible for doctors a few years ago. It has also supported the healthcare centers to reach more people at a time and deliver excellent healthcare service at a minimal cost. The application of big data and cloud computing has also made communication between the patient and doctors more reliable and easier. This resulted in an enhanced patient's engagement in the treatment process with a reduced financial burden on the patient. The considerable impact of IoT, which has been witnessed in recent years, is contributing to the evolution of HIoT applications that includes disease diagnosis, personal care for pediatric and elderly patients, health and fitness management, and supervision of chronic diseases. For a better grasp of these applications, it has been divided into two basic categories, namely, services and applications. The former includes the concepts that are being used while developing an HIoT device and the latter includes the healthcare applications in either diagnosis of a specific health condition or measurements of health parameters. The following sections have included an detailed description of the services and applications of HIoT.

### 4.1. Services

**Figure 3:**
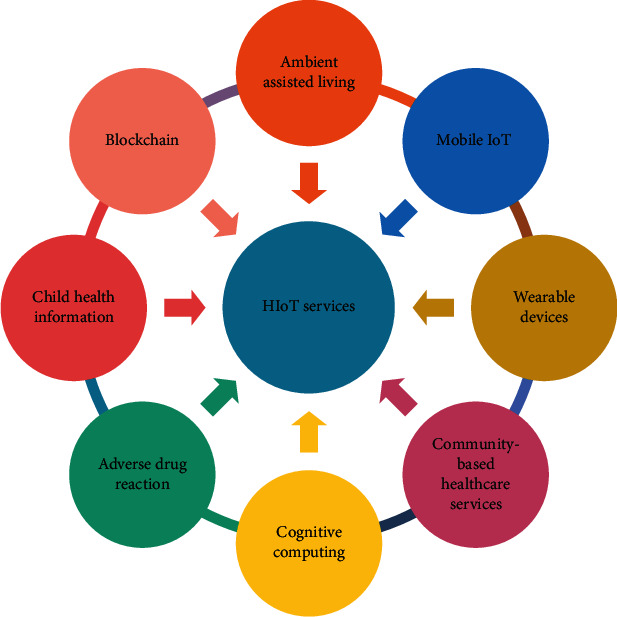
Widely used HIoT services.

Services and concepts have transformed the healthcare industry by providing solutions to various healthcare problems. More services are added day-by-day with a rise in healthcare demands and upgradation of technology. These are now becoming an integral part of designing an HIoT system. Each service in an HIoT environment provides a set of healthcare solutions. The definition of these concepts/services is not unique. The uniqueness of the HIoT systems lies in their applications. Hence, it is hard to outline a generalized definition of each concept. However, to give an insight into the topic, some of the most widely used IoT healthcare services (Figure 3) have been described in the subsequent section.

#### 4.1.1. Ambient Assisted Living

**Figure 4:**
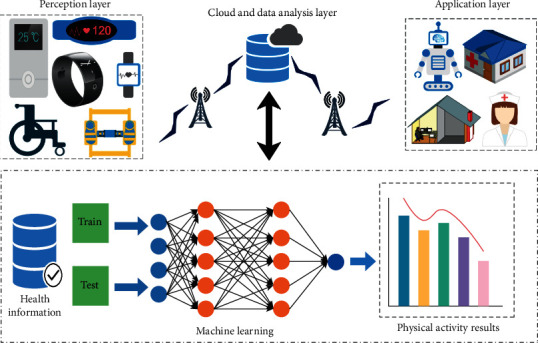
A smart healthcare framework for AAL (reproduced from [27], license no. 496010299387).

Ambient assisted living (AAL) is a specialized branch of artificial intelligence that integrates with IoT and is used for assisting aging people. The main purpose of AAL is to help elderly people to live independently at home with convenience and safety. AAL provides a technique for real-time monitoring of these patients and making sure that they will receive human service-like assistance in case of a medical emergency. This is possible with the engagement of advanced AI technologies, big data analysis, machine learning, and their application in healthcare industries. In general, three basic domains of AAL, namely, activity recognition, environment recognition, and vital monitoring, have been explored by the researchers. However, activity recognition got the utmost interest as it deals with detecting potential threats or emergency health conditions that may affect the well-being of elderly patients. The basic architecture of a smart healthcare framework for AAL is represented in Figure 4. Numerous studies have reported the application of IoT in AAL [28–31]. Shahamabadi [32] proposed a framework that dispenses healthcare solutions to elderly people. The author designed a modular architecture for automation, security, and communication for the AAL. During implementation, IPv6-based low-power wireless personal area networks (6LoWPAN) [33], RFID, and NFC were used as the communication protocol. The device employs a closed-loop communication service to connect the patient with the healthcare providers. The aforesaid architecture was later used as a basis for the development of the more advanced protocol, which can be used to design advanced IoT-based AAL systems (smart objects, devices, and kits). In a recent study, Sandeepa developed an emergency detector for elderly people that assists in monitoring chronic conditions and other potential health-related emergencies. Moreover, the system alerted the caregivers in case of an emergency [34]. IoT-based healthcare systems are now able to track indoor air quality with help of assistive robots. These systems check the quality of air in the environment where the patient resides [35, 36] and trigger alerts to the caregivers when there is a reduction in the air quality below a standard value. In [37], cloud computing has been integrated with IoT to propose a secure, open, and flexible platform for AAL where an IoT-based gateway was employed. The gateway helped in addressing various issues that are associated with security, data storage, and interoperability in the IoT system.

#### 4.1.2. Mobile IoT

**Figure 5:**
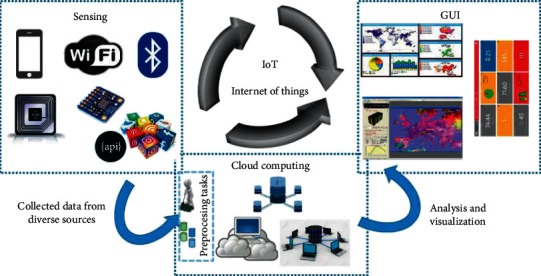
A generalized m-IoT environment (reproduced from [38] under Creative Common License).

Mobile IoT or m-IoT depicts the association of mobile computing, sensors, communication technologies, and cloud computing to track patient's health information and other physiological conditions (Figure 5). In other words, it provides a communication interface between the personal area networks and mobile networks (such as 4G and 5G) to provide an efficient Internet-based healthcare service [33]. The use of mobile has made the HIoT services more accessible to the healthcare practitioner who can access the patient's data, diagnose, and swiftly provide treatment. Several pieces of research have been reported on the application of mobile computing in healthcare [38–40]. Istepanian et al. [41] have developed an m-IoT based system that could monitor the glucose level in diabetic patients which helped in hypoglycemia management. In another study, a mobile gateway-based HIoT system called “AMBRO” was designed where several sensors were used for fall detection and heart rate control. Further, it could locate the patients using an integrated GPS module. In [42], an IoT-based real-time monitoring system has been reported that detects an abnormality in the heart activity and alerts the patient when the heart rate goes beyond 60–100 beats per minute. The security and privacy of the user and user data is an important issue in an m-IoT system. In [43], various methods have been proposed that can be used to address these aforesaid issues including physical and technical safeguards, network security, audit reports, and technical policies.

#### 4.1.3. Wearable Devices

**Figure 6:**
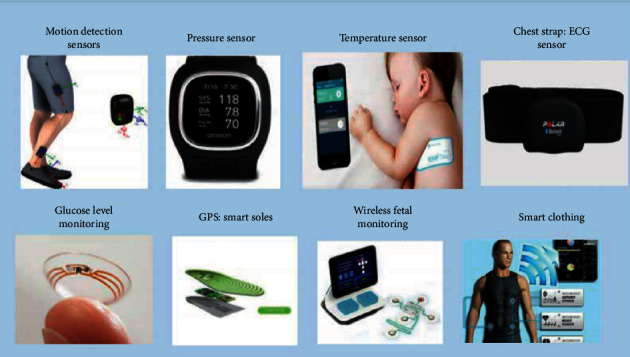
Wearable sensors (reproduced from [27], license no. 496010299387).

Wearable devices help healthcare professionals and patients to deal with various health issues at a reduced cost. These devices are noninvasive and can be developed by integrating various sensors with wearable accessories used by humans such as watch, wristband [44], necklace, shirt, shoes, handbag, caps, and so on [45]. The sensor attached is used to collect the environmental and patient's health information. This information is then uploaded to the server/databases. Some wearable devices are also connected with mobile phones through health applications. Various studies have been reported in the literature showing the use of these wearable devices (Figure 6) and mobile computing in real-time monitoring [46–49]. Castillejo et al. have proposed an activity recognition method by integrating wearable devices in a wireless sensor network for remote monitoring of patients through an e-health mobile application [50]. In a similar study, Jie Wan et al. have developed an IoT-enabled health monitoring device where several sensors (including heartbeat, body temperature, and blood pressure sensors) have been embedded to provide remote health monitoring. Biosignals such as electrocardiograph (ECG) and electromyography (EMG) signals were also analyzed with the help of IoT-enabled wearable systems to extract patient's vital information [51]. The interconnectivity of these wearable devices with a mobile application enhances the computational power of the device. The application can be further used for easy processing and visualization of the collected information.

#### 4.1.4. Community-Based Healthcare Services

Community-based healthcare monitoring is a concept of creating a healthcare network that covers a local community such as a private clinic, a small residential area, a hotel, and so on to monitor the health conditions of the people residing in that area. In a community-based network, various networks are concatenated and can work cooperatively to give a collaborative service. In [52], an IoT-based cooperative medical network was set up to provide healthcare monitoring in remote areas. To establish a secure connection between the networks, different authentication and authorization mechanisms were employed. In another study [51], a community medical network was proposed that was considered as a “virtual hospital.” This helped to provide medical facilities to the needy from a remote location. A resident health network has been proposed in [52]. Herein, a four-layer structural framework was designed for sharing health information that includes medical records of the patients. This information can be accessed by the health centers to provide proper medical advice to the patients who are residing in the locality.

#### 4.1.5. Cognitive Computing

Cognitive computing refers to the process of analyzing a problem the way the human brain does. With recent advancements in sensor technology and artificial intelligence, IoT devices are now integrated with sensors that can mimic the human brain in solving problems. Cognitive computing in an IoT system helps in analyzing hidden patterns that are present in a large volume of data [53]. Further, it enhances the ability of a sensor to process healthcare data and automatically adapt to the surrounding. In a cognitive IoT network, all sensors collaborate with other smart gadgets and provide efficient health services. The use of cognitive computing in an IoT system helps the healthcare providers to make an effective observation of the patient's data and provide proper treatment. In [54], an EEG-based smart healthcare monitoring system has been proposed that uses cognitive computing to decide the pathological condition of the patient. The EEG data, along with other sensor data such as speech, gesture, body movement, and facial expressions, were used to assess a patient's condition. Further, it facilitates emergency help in case of pathogenic conditions. Kumar et al. have proposed a cognitive data transmission method that can effectively detect, record, and analyze patient's health data. During an emergency, the data of the patient, under critical condition, are transmitted with the utmost priority [55].

#### 4.1.6. Adverse Drug Reaction

An adverse drug reaction (ADR) can be characterized as a side effect of taking a medication. The reaction may occur either after a single dose or a long-term administration. This can also be possible due to the adverse reaction when two different medicines are ingested at the same time. ADR does not depend on the type of medicine or the disease and it varies from person to person. In an IoT-based ADR system, a unique identifier/barcode is used to identify each medicine at the patient's terminal [56]. The information about the drug's compatibility with the patient's body can be checked using a pharmaceutical intelligent information system. The information system stores the allergy profile of each patient using e-health records. After analyzing the allergy profile and other vital health information, a decision is made whether the medication is suitable for a patient or not. In a similar study [57], an IoT-based prescription adverse drug event (prescADE) system has been proposed, which can improve patient safety by reducing the ADE.

#### 4.1.7. Blockchain

**Figure 7:**
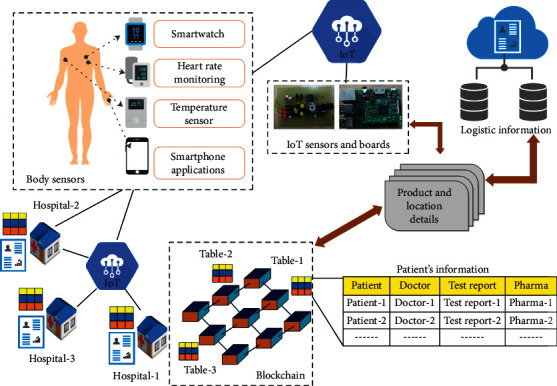
A blockchain-based health monitoring system (modified from [59] under Creative Commons License).

The sharing of data among different medical devices and healthcare providers plays a crucial role in an HIoT network. However, one of the major issues in secure data sharing is data fragmentation. Data fragmentation may lead to a gap in information across healthcare providers, who are associated with a single patient. Insufficient information may hamper the treatment process. Blockchain technology is used to solve the problem of data fragmentation and helps the healthcare centers to establish a connection among the data repositories that are present in the network [58]. This further ensures secure and protective sharing of sensitive medical information and increases transparency between the doctors and patients. Blockchain technology also promotes collaboration among healthcare providers and organizations to do qualitative research (Figure 7). The secure transmission in blockchain technology can be due to three factors. First, it contains an immutable “ledger” that can be accessed and controlled by people. It ensures that once a record is stored in the ledger, it cannot be modified. Further, each transaction in the ledger must follow certain predefined rules. Second, blockchain is a distributed technology and operates simultaneously from multiple devices, computers, etc. Third, blockchain follows the agreement rules and data exchange policies with a smart contract mechanism. The smart contract manages identity and sets out permissions to access different electronic medical reports (EMRs) that are stored in the blockchain. It means doctors are only allowed to go through those EMRs to which they have been permitted. Numerous blockchain projects have been established in the healthcare industry in recent years for the management of EMR, medicine prescription, and clinical pathways [60–62]. Yue et al. have developed an app called healthcare data gateway (HDG) that uses blockchain technology and provides authority to patients to share their information. Herein, the patient can control and share their information without violating the privacy policy [63].

#### 4.1.8. Child Health Information

Child health information (CHI) is a concept that deals with creating awareness for a child's well-being. The main purpose of CHI is to educate and empower children and their parents on the child's overall health including their nutritional values, emotional and mental state, and behavior. The application of IoT has helped researchers to achieve this goal with the development of a platform that can monitor and regulate a child's health. Nigar and Chowdhury have developed an IoT-based framework where a child's mental and physical state can be monitored [64]. Further, necessary measures can be taken with the help of doctors and parents in case of an emergency. In a similar study [65], an IoT-based medical network was developed that connects a medical device with a mobile app. The system collects five different body parameters: height, temperature, SpO_2_, weight, and heart rate. This information is made available to the doctors and health professionals by the app. In [66], the use of an m-health service has been proposed to monitor the food habit of children by the teachers and parents. The app was used to attain good nutritional values in the children.

### 4.2. Applications

**Figure 8:**
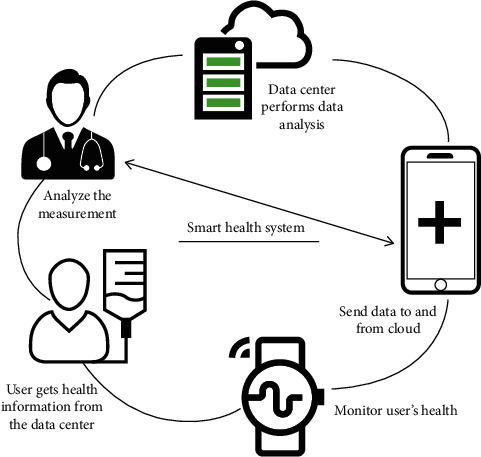
Application of HIoT (reproduced from [40]).

**Figure 9:**
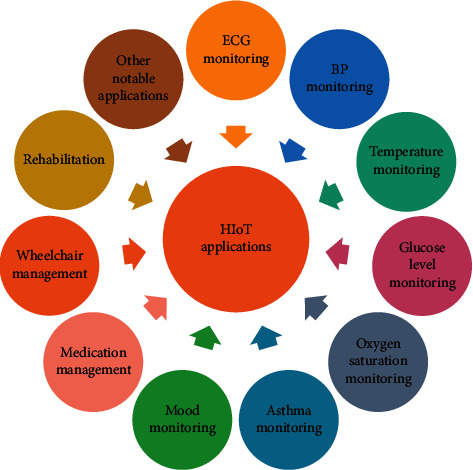
Category of HIoT application.

The HIoT services/concepts are used for the development of different IoT-based applications. Researchers working in the said fields have proposed different concepts to the service of mankind. In simple words, concepts are more developer-centric, whereas applications are user-centric. The rapid development in the IoT-technology has led to the development of more affordable and user-friendly wearable sensors, portable gadgets, and medical devices. These systems can be used to collect patient's information, diagnose diseases, monitor the health of the patients, and generate alerts in case of a medical emergency (Figure 8). In the following section, some of the most recent commercially available devices have been discussed. Further, various HIoT-based applications have been addressed including both single condition and multiple conditions (Figure 9).

#### 4.2.1. ECG Monitoring

Electrocardiogram (ECG) represents the electrical activity of the heart due to the depolarization and repolarization of atria and ventricles. An ECG provides information about the basic rhythms of the heart muscles and acts as an indicator for various cardiac abnormalities. These abnormalities include arrhythmia, prolonged QT interval, myocardial ischemia, etc. The use of IoT technology has found potential application in the early detection of heart abnormalities through ECG monitoring. Numerous studies in the past have employed IoT in ECG monitoring [67–72]. The study reported in [72] has proposed an IoT-based ECG monitoring system that is composed of a wireless data acquisition system and a receiving processor. It employed a search automation method that was used to detect cardiac abnormality in real time. In [73], a small wearable low-power ECG monitoring system was proposed that was integrated with a t-shirt. It used a biopotential chip to collect good quality ECG data. The recorded data were then transmitted to the end-users through Bluetooth. The recorded ECG data could be visualized using a mobile app. The proposed system could be operated with a minimal power of 5.2 mW. Real-time monitoring in an IoT system can be possible after integrating it with big data analytics to manage higher data storage. Bansals and Gandhi have proposed an ECG monitoring system that can handle long-term and continuous monitoring by integrating the concept of nanoelectronics, big data, and IoT [74]. It is worthy to note that in [75], the authors have tried to resolve the issue of power consumption associated with a wearable ECG monitoring system. They have proposed a unique method called compressive sensing that can optimize power consumption and provide optimal performance in ECG monitoring. IoT-based fall detection and ECG monitoring system has also been reported in [76] that uses a cloud-based server and a mobile application. This system was designed to provide real-time monitoring to elderly patients by continuously checking their ECG and accelerometer data.

#### 4.2.2. Glucose Level Monitoring

Diabetes is the condition in which the blood glucose level in the body remains high for a prolonged period. It is one of the most common diseases in humans. Three major types of diabetes are generally found, namely, type-I diabetes, type-2 diabetes, and gestational diabetes. The disease and its types can be identified following three tests, namely, random plasma glucose test, fasting plasma glucose test, and oral glucose tolerance test. However, the most widely used diagnostic method for the detection of diabetes is “fingerpicking” followed by the measurement of blood glucose level. The recent development in IoT technologies has been used in designing various wearable gadgets for blood glucose monitoring that is noninvasive, comfortable, convenient, and safe [77–80]. In [81], m-IoT-based noninvasive glucometer has been proposed for real-time monitoring of blood glucose levels. Herein, the wearable sensors and the healthcare providers were linked through IPv6 connectivity. Alarcón-Paredes et al. have designed a glove for the measurement of blood glucose level that is integrated with a Raspberry Pi camera and a visible laser beam. A set of pictures taken from the fingertip was used for detecting the diabetic condition of the patients [82]. In another study [83], an algorithm based on a double moving average was employed in the IoT architecture for the measurement of the glucose level. It is worth specifying that optical sensors such as infrared LED and near-infrared photodiode have also been used for glucose level measurement. Herein, the light signal reflected from the human body is used to compute the glucose level in the human body [84].

#### 4.2.3. Temperature Monitoring

Human body temperature is an indicator of the maintenance of homeostasis and is an important part of many diagnostic processes. Additionally, a change in body temperature can be a warning sign in some illnesses such as trauma, sepsis, and so on. Keeping track of the change in temperature over time helps the doctors to make inferences about the patient's health condition in many diseases. The conventional way of measuring temperature is using a temperature thermometer that is either attached to the mouth, ear, or rectum. But, the low comfortability of the patient and the high chances of contracting an infection is always an issue with these methods. However, the recent development in IoT-based technologies has proposed various solutions to this problem. In [85], a 3D-printed wearable device was proposed that could be worn on the ear, which tracks the core body temperature from the tympanic membrane using an infrared sensor. The device was integrated with a wireless sensor module and data processing unit. Herein, the measured temperature is not affected by the environment and other physical activities. Gunawan has developed an IoT-based temperature monitoring system using Arduino and Raspberry Pi. The temperature data were stored in the database and were displayed on a web page, which could be accessed through a desktop or a mobile phone [86]. In another study [87], wearable and lightweight sensors were used for real-time measurement of the body temperature in infants. It can also alert the parents whenever there is a rise in temperature above a critical value.

#### 4.2.4. Blood Pressure Monitoring

One of the compulsory procedures in any diagnostic process is the measurement of blood pressure (BP). The most accustomed method of measurement of blood pressure requires at least one person to do the recording. However, the integration of IoT and other sensing technology has transformed the way BP was previously monitored. For example, in [88], a wearable cuffless gadget has been proposed that can measure both systolic and diastolic pressure. The recorded information can be stored in the cloud. Further, the efficiency of this device was tested on 60 persons and the accuracy was validated. Guntha has implemented cloud computing and fog computing in the IoT-based BP measurement system [89]. This prepared the system for long-term real-time monitoring. The device could also store the recorded data for future references. In a similar study [90], a deep learning-based CNN model with time-domain characteristics was used for the evaluation of systolic and diastolic blood pressure. The measurement of BP using the ECG signal and photoplethysmogram (PPG), recorded from the fingertip, has been proposed in [91]. Herein, the BP was computed using the attached microcontroller module and then the recorded data were sent to the cloud storage.

#### 4.2.5. Oxygen Saturation Monitoring

Pulse oximetry is the noninvasive measurement of oxygen saturation and can be used as a vital parameter in healthcare analysis. The noninvasive method eliminates the issues related to the conventional approach and provides real-time monitoring. The advancement in the pulse oximetry that comes from the integration of IoT-based technology has shown potential application in the healthcare industry. In [92], a noninvasive tissue oximeter was proposed that could measure the blood oxygen saturation level, along with heart rate, and pulse parameters. Further, the recorded information could be transmitted to the server using various communication technologies such as Zigbee or Wi-Fi. Based on the recorded data, a medical intervention decision was made. In another study [93], an alarm system that can alert the patients when the oxygen saturation reaches a critical level was reported. The system was integrated with a pulse oximeter and WLAN router that were connected using the Blynk server. Moreover, Von Chong et al. have proposed a multispectral sensor that reduces the adverse effect of a single LED [94]. A low-power and cost-effective remote patient monitoring system has been proposed in [95]. The device can be effectively used for real-time monitoring.

#### 4.2.6. Asthma Monitoring

Asthma is a chronic illness that can affect the airways and may cause difficulty in breathing. In asthma, the airways shrink due to the swelling of the air passage. This follows many health issues such as wheezing, coughing, chest pain, and shortness of breath. There is no suitable time for an asthma attack to come, and an inhaler or nebulizer is the only lifesaver at that moment. Hence, there is a potential need for real-time monitoring of this condition. Numerous IoT-based systems for asthma monitoring have been proposed in recent years [96–98]. In [99], a smart HIoT solution for asthma patients was proposed that was used to record respiratory rate using a smart sensor. The health information was stored in a cloud server that gives access to caregivers for diagnostic and monitoring purposes. Raji proposed a respiratory monitoring and alarm system where an LM35 temperature sensor was used to measure the respiratory rate [100]. This was achieved by monitoring the temperature of the inhaled and exhaled air. The respiration data were sent to the health center and were displayed on a web server. The proposed system also triggered an alarm and automatically sent a message to the patient once a threshold value was reached. In another study [101], the proposed system not only monitored and warned the patients about the asthma condition but also suggested the patients about the right amount of the medication to be administered. Further, the system was capable to analyze the environmental conditions and direct the patient to move from a place that is not suitable for his health. Machine learning, cloud computing, and big data analysis techniques have also been integrated with IoT-based devices to effectively track asthma [102, 103]. A list of features that can be added while thinking about future development in the IoT-based asthma monitoring system has been proposed in [104]. Some of the most potential features include peak flows, pollen, humidity, air temperature, and asthma symptoms.

#### 4.2.7. Mood Monitoring

Mood tracking provides vital information regarding a person's emotional state and is used to maintain a healthy mental state. It also assists healthcare professionals while dealing with various mental diseases such as depression, stress, bipolar disorder, and so on. Self-monitoring of the emotional state enhances a person's understanding of their mental condition. In [105], a mood mining approach was reported that uses a CNN network to evaluate and categorize a person's mood in 6 categories: happy, excited, sad, calm, distressed, and angry. In a similar study [106], the real-time mood measurement was achieved using an interactive system called “Meezaj.” The app also showed the importance of happiness in decision making and assists the policymaker in identifying those important factors that play a crucial role in defining a person's happiness. With the integration of an advanced machine learning algorithm, now stress can be detected beforehand with the help of heartbeat rate. Further, the system can communicate with the patient about their stress condition [107]. It is interesting to note that the analysis of the stress condition can also be useful in designing an IoT-based system that can prevent an accident. Jasleen et al. have proposed a wearable device that can estimate four negative emotions/moods (anger, stress, terror, and sadness) of a person/driver. By analyzing the variation in these emotions, the intelligent system decides whether the driver is in a subconscious state or not. The system stops the dc motor of the vehicle once a driver achieved the subconscious state.

#### 4.2.8. Medication Management

Medication adherence is a common issue in the healthcare industry. Nonadherence to the medication schedule may increase the adverse health complications in patients. Medication nonadherence is mostly found in elderly people as they develop clinical conditions like cognitive decline, dementia, and so on as the age progresses. Hence, it is difficult for them to strictly follow the prescriptions of doctors. Numerous research in the past has focused on tracking the patient's compliance with medication through the application of IoT [108–111]. In [112], a smart medical box was developed that can remind people of their medication. The box has three trays where each tray contains the medicine for three different times (morning, afternoon, and evening). The system also measures some of the vital health parameters (blood glucose level, blood oxygen level, temperature, ECG, and so on). All the recorded data are then sent to the cloud server. A mobile app was used to establish communication between the two end-users. The recorded information can be accessed by doctors and patients using the mobile app. In another study [113], the information about the storage condition of the medicine such as temperature and humidity was also recorded. This helped the patients to maintain the required storage environment. One of the more specific examples of medication management is “Saathi” [114]. This pill monitoring system was specifically designed for the woman going through in vitro fertilization (IVF) treatment. Since the IVF process demands a strict medication schedule, the proposed device gives women the facility to remind their daily medication and injections, track real-time medicine consumption, and communicate with the healthcare providers. Moreover, an adaptive IoT-based smart medication system was reported in [115] that uses fuzzy logic to analyze the data collected from the temperature sensor. The system is efficient to treat fever by continuous monitoring of body temperature and then automatically adjust the time and dose of medicine during treatment.

#### 4.2.9. Wheelchair Management

A wheelchair is an inseparable part of the life of patients with restricted mobility. It provides them physical as well as psychological support. However, the application of a wheelchair is limited when the disability is due to brain damage. Hence, new research is focusing on integrating the navigation and tracking system with these wheelchairs. IoT-based systems are now showing potential results in achieving this goal [116–119]. In [120], an IoT-based steering system, integrated with a real-time obstacle avoidance system, has been proposed. The steering system can detect obstacles by employing image processing techniques on the recorded real-time videos. The use of mobile computing has enabled wheelchair management to be more interactive and easy for patients. A smart wheelchair as represented in [121] was developed by the integration of various sensors, mobile technologies, and cloud computing. The system includes a mobile app that can help the patients to interact with the wheelchair and the caregivers. The app also enables caregivers to monitor the wheelchair from a distance. In another study [122], an IoT-based wheelchair monitoring system has been proposed that used hand gestures for controlling the wheelchair. The designed model is especially applicable for patients having quadriplegia. The hand gesture information was recorded using the RF sensor that was present in the hand gloves and was used to control the wheelchair. Further, the sensor data were transmitted to the server and could be stored in the cloud. The doctors/caregivers can access the data from the cloud and can use this information for diagnosis. It is worth specifying that in [123], a more advanced and automated smart wheelchair was reported that not only monitored the wheelchair movement but also provided an umbrella, foot mat, head mat, and obstacle detection features. Herein, the designed system provided more efficient interaction with the living environment.

#### 4.2.10. Rehabilitation System

Physical medicine along with rehabilitation is effective in restoring the functional ability of a patient with a disability. Rehabilitation involves identifying the problem and helping the patients to regain their normal life. The application of IoT in rehabilitation is diverse and can be seen in the treatment of cancer, sports injury, stroke, and other physical disabilities [124–127]. A smart walker rehabilitation system has been proposed in [128] that used a multimodal sensor to monitor the walking pattern of the patient and evaluate the movement metrics. When a patient used the smart walker, it measured different movement matrices such as orientation angle, elevation, force, and so on. A mobile app was used by the doctors to access these data and to provide diagnostic reports. Moreover, a stroke rehabilitation system was developed by integrating a smart wearable armband, robotic hand, and machine learning algorithm [129]. The armband was designed using a low-power IoT-based textile electrode that can measure, preprocess, and transmit the biopotential signal. Further, the 3D-printed robotic arm analyzed the muscle activity and assisted the patient to correct their motion pattern during the after-stroke recovery period. In another study, a sports rehabilitation system was reported that monitored temperature, motion posture, electromyography (EMG), electrocardiography (ECG), and so on and provided feedback to the athletes. The recorded information could be used by healthcare professionals to predict the patients' recovery and formulate rehabilitation programs.

#### 4.2.11. Other Notable Applications

The application of HIoT is disparate and not limited to the aforesaid functions. With the rapid growth of technology, the number of HIoT applications is increasing significantly. Some of the research areas where the integration of IoT devices was not explicitly demonstrated previously are now using this technology efficiently. This may include cancer treatment, remote surgery, abnormal cellular growth, hemoglobin detection, etc. In [130], a new IoT-based framework for cancer treatment was proposed that integrated various stages of cancer treatment including chemotherapy and radiotherapy. A mobile app was used for online consultation from the doctors. The lab-test results of patients were stored in the cloud server and could be accessed by the healthcare provider to decide the time and dosage of medication. Another potential application is the detection of lung cancer using various state-of-the-art machine learning algorithms with an IoT-based system [131–133]. Moreover, a recent piece of research also suggested the detection of skin lesions using an IoT-based system [134]. Cecil et al. have employed IoT in designing the next-generation surgical training framework [135]. The device used virtual reality to develop a training environment and also provided a platform to interact with other surgeons from different locations. In [136], a human-robot collaborative system has been proposed that can effectively perform minimally invasive surgery. Using a portable device, the hemoglobin level in the blood can be monitored [137]. The device employed photoplethysmography (PPG) sensors, a light-emitting diode (LED), and photodiodes for the measurement of hemoglobin. The efficiency of the device was further validated by comparing the results with the established colorimetric test.

## 5. Challenges, Limitations, and Future Scope

In the last few years, the healthcare industry has witnessed remarkable technological development and its application in solving healthcare-related issues. This has significantly improved the healthcare services, which have now been brought at the fingertip. With the application of smart sensors, cloud computing, and communication technologies, IoT has successfully revolutionized the healthcare industry. Like other technologies, IoT also has certain challenges and issues that provide potential scope for future research. Some of the issues have been discussed in the subsequent section.

### 5.1. Servicing and Maintenance Cost

Of late, there are rapid technological advancements that would require continuous upgradation of the HIoT-based devices from time to time. Every IoT-based system involves a large number of connected medical devices and sensors. This involves high maintenance, servicing, and upgradation costs that may impact the financials of not only the company but also the end-users. Hence, the inclusion of sensors that can be operated with a lower maintenance cost is required.

### 5.2. Power Consumption

Most of the HIoT devices run on battery. Once a sensor is put on, the replacement of the battery is not easy. Hence, a high-power battery was used to power such a system. However, currently, researchers worldwide are trying to design healthcare devices that can generate power for themselves. One such potential solution may be the integration of the IoT system with renewable energy systems. These systems can help in alleviating the global energy crisis to a certain extent.

### 5.3. Standardization

In the healthcare industry, a large number of vendors are manufacturing a varying range of products. Most of these products claim to follow standard rules and protocols in the design process. However, there is a lack of validity. Hence, the construction of a dedicated group is required that can standardize these HIoT devices based on the communication protocols, data aggregation, and gateway interfaces. The validation and standardization of electronic medical records (EMRs) recorded by the HIoT devices are also to be considered extensively. This can be achieved when various organizations and standardization bodies such as Information Technology and Innovation Foundation (IETF), the European Telecommunications Standards Institute (ETSI), the Internet Protocol for Smart Objects (IPSO), and so on can collaborate with the researchers to form working groups for the standardization of the devices.

### 5.4. Data Privacy and Security

The integration of cloud computing has transformed the idea of real-time monitoring. But, this also has made healthcare networks more vulnerable to cyberattacks. This may lead to mishandling of patients' valuable information and may affect the process of treatment. To prevent an HIoT system from this malicious attack, several preventive measures must be taken while designing a system. The medical and sensing devices included in an HIoT network must evaluate and employ identity authentication, secure booting, fault tolerance, authorization management, whitelisting, password encryption, and secure pairing protocols to avoid an attack. Similarly, the network protocols such as Wi-Fi, Bluetooth, Zigbee, and so on must be integrated with secured routing mechanisms and message integrity verification techniques. Since IoT is a connected network where each user is linked to the server, any glitch in the security services of IoT may compromise the privacy of the patient. This could be fixed with the creation of a more secure environment through the integration of advanced and protected algorithms and cryptographies.

### 5.5. Scalability

Scalability represents the ability of a healthcare device that can adapt to the changes in the environment. A system with higher scalability works smoothly without any delay and makes efficient use of the available resources. Hence, it is crucial to design a device with higher scalability. This further makes a system more efficient for present and future uses. An HIoT system is the interconnection of different medical devices, sensors, and actuators, which are used to share information through the Internet. The lack of uniformity among the connected devices of an HIoT system decreases the scalability of the system and hence must be managed efficiently.

### 5.6. Identification

Healthcare professionals deal with multiple patients and caregivers at the same time. Similarly, when a patient deals with multiple health issues, he interacts with multiple doctors. Thus, it is crucial to exchange the identity of the patient, caregiver, and doctors among each other during a single treatment process to avoid confusion and maintain the smooth functioning of the healthcare system.

### 5.7. Self-Configuration

The IoT devices must give more power to the users by including the feature like manual configuration. This will enable the users to change the system parameters according to the application demand and also with the change in the environmental conditions.

### 5.8. Continuous Monitoring

Many healthcare situations demand long-term monitoring of the patient during treatment as in the case of chronic diseases, heart diseases, etc. In such situations, the IoT device must be able to perform real-time monitoring efficiently.

### 5.9. Exploration of New Diseases

With the rapid growth in mobile technology, new healthcare apps are added with passing days. Though a large number of mobile apps are available for healthcare applications, the types of diseases for which these apps were designed are still limited. Hence, there is a need to include more diseases that were either neglected or got inadequate consideration in the past. This will add up to the diversity of the HIoT applications.

### 5.10. Environmental Impact

The development of an HIoT system requires the integration of various biomedical sensors with semiconductor-rich devices. The manufacturing and fabrication mostly require the use of earth metal and other toxic chemicals. This may create an adverse effect on the environment. Hence, a proper regulatory body must be created to control and regulate the manufacturing of the sensors. Further, more research must be devoted to making sensors using biodegradable materials.

## 6. Conclusion

The current review investigated different aspects of the HIoT system. Comprehensive knowledge about the architecture of an HIoT system, their component, and the communication among these components has been discussed herein. Additionally, this paper provides information about the current healthcare services where the IoT-based technologies have been explored. By employing these concepts, the IoT-technology has helped healthcare professionals to monitor and diagnose several health issues, measure many health parameters, and provide diagnostic facilities at remote locations. This has transformed the healthcare industry from a hospital-centric to a more patient-centric system. We have also discussed various applications of the HIoT system and their recent trends. Further, the challenges and issues associated with the design, manufacturing, and use of the HIoT system have been provided. These challenges will form a base for future advancement and research focus in the upcoming years. Moreover, a comprehensive up-to-date knowledge on the HIoT devices has been provided for the readers who are not only willing to initiate their research but also make advancements in the said field.

## Data Availability

No data were used to support this study.
